# Chemically Roughened, Sputtered Au Films with Trace-Loaded Manganese Oxide for both On-Chip and Off-Chip High Frequency Supercapacitors

**DOI:** 10.3390/nano11020257

**Published:** 2021-01-20

**Authors:** Pai Lu, Haitao Xue, Wentao Liu, Zhongbao Feng, Qiang Sun

**Affiliations:** School of Metallurgy, Northeastern University, Shenyang 110819, Liaoning, China; xuehaitao2020@163.com (H.X.); wentaoliu2021@163.com (W.L.); fengzb@smm.neu.edu.cn (Z.F.); sunq@smm.neu.edu.cn (Q.S.)

**Keywords:** high frequency supercapacitor, thin film electrode, sputtering, oxidative etching, chemical roughening, trace loading

## Abstract

High frequency supercapacitors (HFSCs) are promising in alternating current line filtering and adaptable storage of high-frequency pulse electrical energy. Herein, we report a facile yet integrated-circuit-compatible fabrication of HFSC electrodes by combining chemical roughening of the sputtered metal (Au) films and in situ trace loading of a pseudocapacitive material (MnO*_x_*). The developed electrode fabrication route is versatile for different substrates, and is described with the application paradigms of both on-chip (with Si/SiO_2_ substrate) and off-chip (without Si/SiO_2_ substrate, with Ti substrate as an example in this study) HFSCs. With Au/MnO*_x_* films on Si/SiO_2_ substrates as the working electrodes, the derived on-chip HFSC displayed satisfactory performance in high frequency applications (i.e., an areal capacitance of 131.7 µF cm^−2^, a phase angle of −78°, and a RC time constant of 0.27 ms, at 120 Hz).

## 1. Introduction

Supercapacitors have been widely pursued due to their fast charging/discharging capabilities, which readily complement the electrochemical batteries used in high power applications [1,2]. Supercapacitors respond well in time scales of seconds [3]; however, when a very high frequency is applied (e.g., above 100 Hz), most commercial supercapacitors behave more like resistors than capacitors due to their large internal resistance [4]. High frequency supercapacitors (HFSCs), i.e., supercapacitors which are required to handle high frequencies, were first reported in 2010 by Miller et al., who proposed their use to filter alternating current (AC) at 120 Hz, replacing conventional aluminum electrolytic capacitors (AECs) [5]. Thanks to their much higher capacitance compared to AECs, the application of HFSCs is expected to facilitate miniaturization. In recent years, HFSCs which respond well at above 100 Hz and even up to several thousands of Hz have been shown to effectively collect the pulse energy generated by renewable energy harvesters, outperforming all other electrochemical energy storage devices [6]. Motivated by the aforementioned potential for AC filtering and effective storage of high frequency pulse energy, HFSCs have been positioned at the frontier of research [7].

Due to the requirement of high frequency response, the design of HFSC electrodes is required to enable both superior electronic and ionic transport [8]. During the past decade, electrical double layer capacitive nanocarbons (graphene (131 µF cm^−2^ at 120 Hz) [9], carbon nanotubes (CNT, 601 µF cm^−2^ at 120 Hz) [10], porous carbon (132 µF cm^−2^ at 120 Hz) [11]), and pseudocapacitive materials (conductive polymer (270 µF cm^−2^ at 120 Hz) [12], MXene (300 µF cm^−2^ at 120 Hz) [13]) have been explored as electrode materials for HFSCs. Various proposed electrode designs (typically, vertically aligned graphene [14], a thin layered porous CNT framework [10], macroporous carbon [11], conductive polymer thin films [12], and MXene/CNT composite films with incorporated abundant pores [15]) have been shown to attain great high frequency response. However, these electrode fabrication routes involve high temperature processing (e.g., chemical vapor deposition of graphene at 1000 °C, thermal transformation of precursors at 800–900 °C for macroporous carbon film preparation), or tedious, multistep wet chemical preparation (e.g., etching and purification of graphene or MXene sheets, followed by multistep solution filtration and troublesome film transfer), which are obstacles to large scale application and commercialization.

The R&D of HFSCs is mainly aiming at enabling their application in electronics. Most recently, their potential application in the Internet of Things (IoTs) to power fast data transfer has aroused immense interest [16]. From this perspective, the development of a facile yet integrated-circuit (IC)-compatible electrode fabrication strategy [17,18] is of significance for deriving circuit-integratable HFSCs and facilitating the realization of miniaturized and smart electronics. The currently available, laborsome strategies apparently cannot meet this demand. Herein, we propose a novel HFSC electrode fabrication strategy based on room temperature etching of sputtered Au film and in situ trace loading of capacitive manganese oxide. The whole production process could be readily implemented in the IC industry. Also, for the first time, we show that abundant pseudo-capacitive metal oxides can be employed in HFSCs as active materials when the electrode is appropriately designed.

## 2. Experimental

### 2.1. HFSC Electrode Fabrication

#### 2.1.1. Au Film Growth

To build on-chip and off-chip HFSCs, commercially available Si/SiO_2_ wafers (Silicon Materials, Kaufering, Germany) and Ti foils (Sinopharm Chemical Reagent Co., Ltd., Beijing, China) were respectively used as building substrates.

Au film growth on Si/SiO_2_ wafers: 50 nm Cr films as the buffer layers were first deposited onto Si/SiO_2_ wafers by electron beam evaporation to enhance the adhesion. During the deposition, the base pressure range and working pressure range were maintained at 5 × 10^−7^ to 1 × 10^−6^ Torr, and 1 × 10^−6^ to 5 × 10^−6^ Torr, respectively. A sensor-based digital system allowed precise control over the deposition rate, i.e., around 0.04 nm s^−1^. Afterwards, Au films with a thickness of 100 nm were deposited via room temperature DC sputtering at 35 W. The deposition rate was controlled at around 0.06 nm s^−1^ by a sensor-based digital system.

The same Au DC sputtering process was applied for Au film growth on Ti foils, except for that no buffer layers were employed.

#### 2.1.2. Oxidative Etching

The sputtered Au films on different substrates were exposed to 0.02 M KMnO_4_ solution for 14–16 h at room temperature. Afterwards, the obtained electrodes were cleaned with deionized water and dried in N_2_.

### 2.2. HFSC Device Assembly

Two identical electrodes were assembled in a typical sandwich configuration, with glass fiber as the separator and 1 M Na_2_SO_4_ as the electrolyte. Parafilm was used to package the device to avoid leakage of the electrolyte.

### 2.3. Material Characterization and Electrochemical Measurement

A scanning electron microscope (SEM) (FEI Quanta250FEG, Hilsboro, OR, USA) and atomic force microscope (AFM) (Bruker MultiMode 8, Billerica, MA, USA) were used to characterize the morphologies and surface roughness. An X-ray photo-electron spectroscopy (XPS) (Thermo Scientific ESCALAB 250 system, Waltham, MA, USA) measurement was performed to analyze the chemical elements.

Three-electrode measurements and testing of the full cells were conducted using a CHI 660E potentiostat (CHI, Shanghai, China). For cyclic voltammogram (CV) measurements, the electrochemical windows were maintained at 0–0.8 V relative to Ag/AgCl (saturated KCl) reference electrode. Electrochemical impedance spectroscopy (EIS) was measured with an amplitude of 5 mV at the open circuit potential.

## 3. Results and Discussion

**Figure 1:**
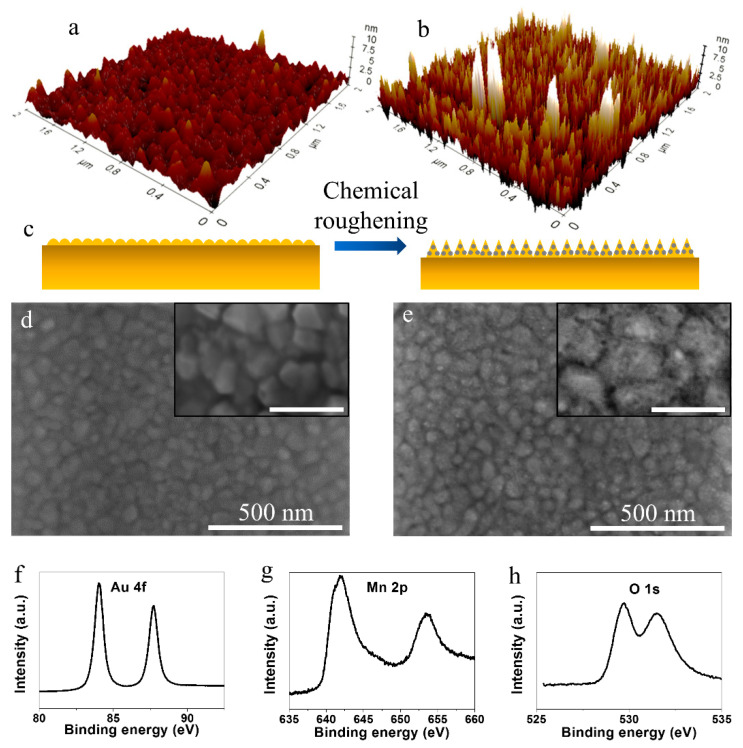
AFM characterization of sputtered Au film on Si/SiO_2_ (**a**) before and (**b**) after oxidative etching. (**c**) Sketch of the electrode formation via oxidative etching and in situ trace loading of MnO*_x_*. SEM characterization of (**d**) P-Au and (**e**) T-Au/MnO*_x_* (inset scale bar is 100 nm). XPS characterization of T-Au/MnO*_x_* electrode for (**f**) Au 4f orbit, (**g**) Mn 2p orbit, and (**h**) O 1s orbit.

The proposed electrode fabrication was inspired by the oxidative etching of Au by KMnO_4_ [19]. The slow oxidation of Au^0^ to soluble Au^3+^ resulted in the chemical roughening of the sputtered Au films. Figure 1a,d show that the DC sputtering deposited a pristine Au film (P-Au), comprising Au nano-islands with the lateral size ranging from 10 to 40 nm, according to the classical island growth mode, and a maximum surface roughness of 4 nm. The etching of Au nano-islands was revealed by the AFM characterization of the chemically treated Au (T-Au) film (Figure 1b), which clearly showed that the surface roughness had been greatly enhanced after exposing the sputtered P-Au to KMnO_4_ solution, with a maximum surface roughness of 15 nm. Figure 1e shows that the oxidative etching preferentially took place at the grain boundary sites, which led to the formation of deeper trenches between the Au nano-islands, representing enhanced roughness. Accompanied with the oxidative etching, the concurrent reduction of KMnO_4_ resulted in the formation of manganese oxide (MnO*_x_*) in situ. Benefiting from the slow redox rate of Au and KMnO_4_, a trace loading of MnO*_x_* onto the Au nano-islands could be realized. The inset in Figure 1e shows that very tiny MnO*_x_* nano-units were sparsely distributed on the Au islands. Figure 1c describes the process schematically. The trace MnO*_x_* product was examined by XPS (Figure 1f–h). The Au 4f spectrum (Figure 1f) with two peaks at 83.9 eV (Au 4f_7/2_) and 87.6 eV (Au 4f_5/2_) corresponded to the Au^0^ substrate [20]. The formation of MnO*_x_* was confirmed by the XPS signals from the Mn 2p (Figure 1g) and O 1s (Figure 1h) orbits. The peaks with binding energies of 642.1 eV and 653.5 eV were attributed to the Mn 2p_3/2_ and Mn 2p_1/2_ spin-orbit doublets, respectively, for MnO_2_, which demonstrated that the main valence state of MnO_x_ was IV [21]. Inferred from the O 1s spectrum, Mn−O−Mn (529.6 eV) in manganese oxide, and Mn−O−H (531.4 eV) in manganese hydroxide had both been incorporated [22].

**Figure 2:**
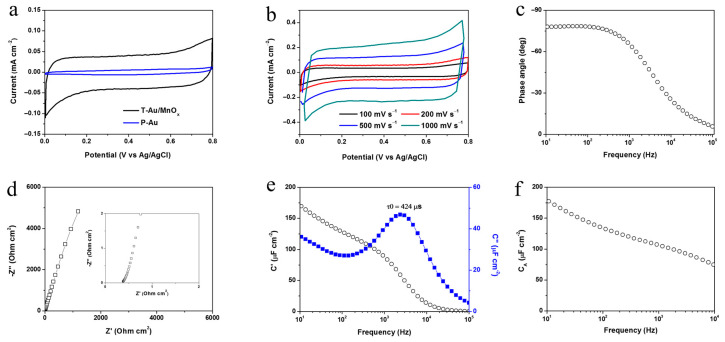
Three-electrode measurement: (**a**) CV comparison of P-Au and T-Au/MnO*_x_* at 100 mV s^−1^, (**b**) CV measurements of T-Au/MnO*_x_* at varying scan rates. Full cell measurement of on-chip HFSCs: (**c**) Phase angle versus frequency, (**d**) Nyquist plot, (**e**) Plots of the real or imaginary part of the specific capacitance as a function of frequency, (**f**) Areal specific capacitance versus frequency.

From the CV comparison result in the three-electrode test (Figure 2a), it was found that the T-Au electrode with loaded trace MnO*_x_* displayed typical capacitive behavior and much larger integrated capacitive current than P-Au; this was attributed to the increased number of electrochemical active sites for electrical double layer capacitance via surface roughening and the grown pseudo-capacitive MnO*_x_* on the Au nano-islands. An areal capacitance of 380 µF cm^−2^ was achieved at 100 mV s^−1^ (capacitance calculation, presented in Supplementary Materials). Figure 2b indicates that an ideal, rectangular shape of CV curves could be maintained in a wide range of CV sweeping rates in the three-electrode test. With the T-Au/MnO*_x_* on Si/SiO_2_ substrates as the electrodes, the electrochemical performance of the on-chip full cell was studied. Generally, the phase angle at 120 Hz is significant for 60 Hz AC response. The assembled on-chip full cell showed a phase angle of −78° at 120 Hz (Figure 2c), which was very close to that of commercial AECs for AC filtering (typically, with a phase angle of around −80° at 120 Hz [12]), indicating its high frequency response capability. The resistor-capacitor (RC) time constant (τ_RC_) was calculated to be 0.27 ms, i.e., much smaller than 8.3 ms (for 120 Hz filtering). This τ_RC_ also confirmed its promising high frequency handling capability. The measured Nyquist plots (Figure 2d) revealed a low equivalent series resistance (ESR) of the on-chip HFSC (0.38 Ω cm^2^). To the best of our knowledge, this is the lowest ESR of any supercapacitor based on nonconductive pseudocapacitive active materials to date. The low ESR is beneficial for the fast response; we attributed this to two aspects: (1) a very good chemical bonding between MnO*_x_* and Au, realized by the redox reaction (between Au and KMnO_4_) -promoted deposition, and (2) the sparse loading of very tiny MnO*_x_* nano-units facilitating electrolyte access and assisting in the reduction of the material resistance by shrinking. The specific real and imaginary capacitances of this on-chip HFSC are plotted versus frequency in Figure 2e. Given the frequency (f_0_ = 2359 Hz) at the maximum C″, the relaxation time constant τ_0_ (= 1/f_0_) was derived, i.e., 424 µs, which was the minimum time to discharge its maximum energy with an efficiency ≥ 50% [23]. High f_0_ and small τ_0_ are also indicators of fast frequency response. The τ_0_ of our on-chip HFSC was much smaller than those of commercial supercapacitors based on activated carbon (>200 ms) [24]. The area capacitance as a function of frequency of this HFSC is presented in Figure 2f. At 120 Hz, the HFSC was capable of delivering an areal capacitance of 131.7 µF cm^−2^, which is comparable with most nanocarbon-based HFSCs [4,5,6,9,10,11]; however the electrode fabrication technique used in the present study is much easier to scale up. The areal capacitance can be kept at around 100 µF cm^−2^, even when the on-chip HFSC is operated at up to thousands of Hz. Therefore, the proposed HFSC demonstrates promising potential to collect pulsed energy from various renewable energy harvesters with extremely high frequency output [4].

**Figure 3:**
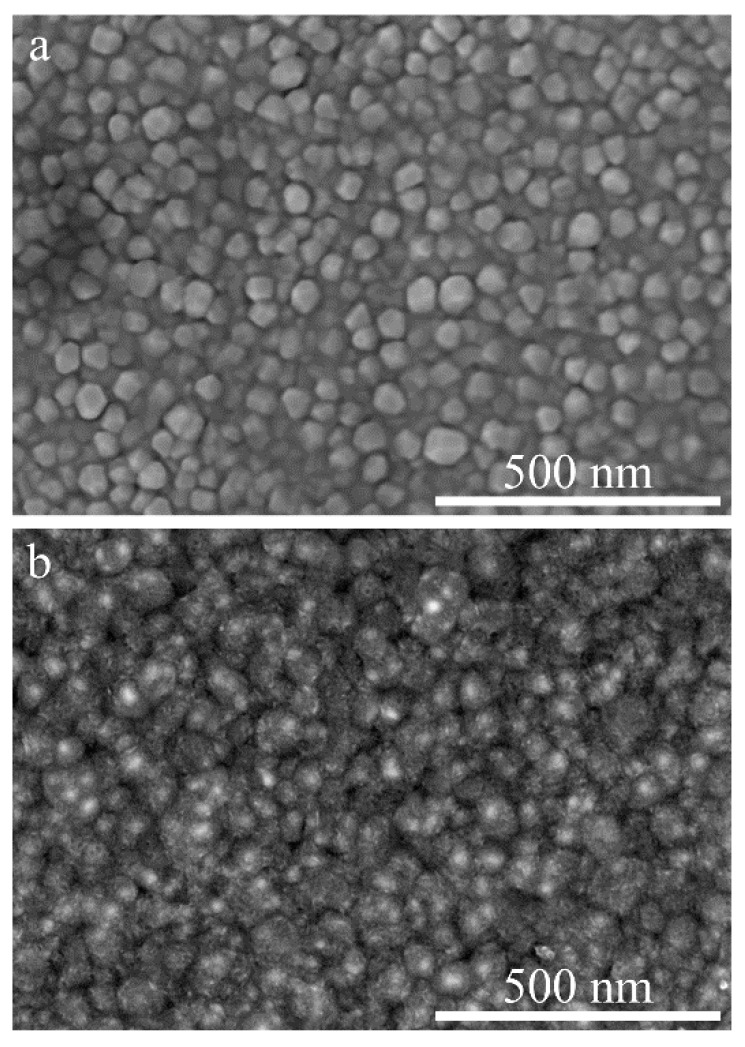
SEM characterization of sputtered Au film on Ti foil (**a**) before and (**b**) after the oxidative etching.

**Figure 4:**
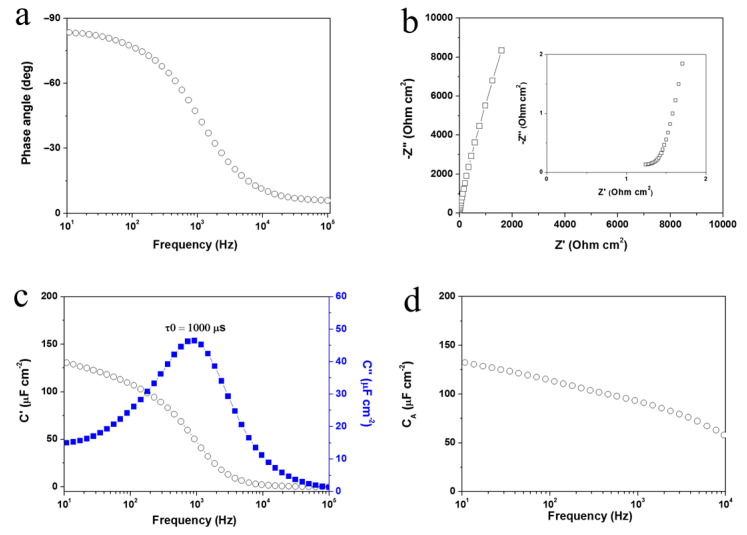
Full cell measurement of off-chip HFSCs with Ti foils as the substrates: (**a**) Phase angle versus frequency, (**b**) Nyquist plot, (**c**) Plots of the real or imaginary part of the specific capacitance as a function of frequency, (**d**) Areal specific capacitance versus frequency.

To prove the versatility, i.e., in terms of the choice of substrate, of the proposed electrode fabrication technique, we described an off-chip HFSC with the KMnO_4_-treated Au films on Ti substrates as the electrodes. Figure 3 shows the morphologies of the Au/Ti film before and after the oxidative etching. The trace-loading of MnO*_x_* was confirmed in the same way as that of the on-chip HFSC electrodes. The high frequency response capabilities of the assembled off-chip HFSC are presented in Figure 4, with a phase angle of −77° at 120 Hz (Figure 4a), and an ESR of 1.24 Ω cm^2^ (Figure 4b). The τ_0_ derived at f_0_ in Figure 4c was 1000 µs. An areal capacitance of 112.2 µF cm^−2^ at 120 Hz was achieved. All the performance indicators indicated the high frequency handling capability of this off-chip HFSC. Compared with existing on-chip HFSCs, the off-chip HFSC exhibited a slightly larger ESR, which may have been due to the difference in substrate geometry. Commercial Ti foil without any surface modification is not as flat and smooth as Si wafer. The deposition of a 100 nm thick Au film on a coarse Ti foil could lead to the formation of cracks and local discontinuity within the Au film, or even cavities between Au and the underlying Ti substrate. As a result, the electron transfer pathway could be prolonged, and Au/Ti interphase resistance could be generated. All these factors are obstacles to lowering the ESR. It is expected that this problem may be overcome by surface treatment of the metal substrates.

## 4. Conclusions

In this study, we developed a facile, room temperature fabrication technique for HFSC electrodes. A slow redox reaction between Au and KMnO_4_ is the basis for deriving enhanced HFSC performance via chemical roughening of sputtered Au films to create increased surface area, and trace loading of MnO*_x_* nano-units as the active material to contribute to pseudo-capacitance as well as reduce the material resistance by size shrinking. The DC sputtering of Au films and subsequent oxidative etching could be readily implemented in the IC industry. The reported electrode fabrication technique is also versatile, i.e., in terms of the choice of substrate, which demonstrates its wide adaptability.

## Supplementary Materials

The detailed capacitance calculation is available online at https://www.mdpi.com/2079-4991/11/2/257/s1.
