# Supplementary material for: Chemically Roughened, Sputtered Au Films with Trace-Loaded Manganese Oxide for both On-Chip and Off-Chip High Frequency Supercapacitors
Source: Nanomaterials (Basel). 2021 Jan 20;11(2):257. doi: 10.3390/nano11020257 (PMC7909291; doi:10.3390/nano11020257)
Supplement: Supplementary file 1 [file nanomaterials-11-00257-s001.pdf]

Supplementary Materials: Chemically Roughened, Sputtered Au Films with Trace-Loaded Manganese Oxide for both On-Chip and Off-Chip High Frequency Supercapacitors

Pai Lu *, Haitao Xue, Wentao Liu, Zhongbao Feng and Qiang Sun

Calculation:

Three electrode measurement:

The areal specific capacitance (C_A) based on CV is calculated by:

$$C_A = \frac{\int i(V)dV}{2A\Delta V\upsilon}$$

where $\int i(V)dV$ is the integrated area of the CV curve, and A, ΔV, υ, are the electrode area, working potential range, scan rate.

Two electrode full cell measurement:

The areal specific capacitance (C_A) is calculated by:

$$C_A = -\frac{1}{2\pi f Z'' A}$$

The resistor-capacitor time constant (τ_{RC}) is calculated by:

$$\tau_{RC} = -\frac{Z'}{2\pi f Z''}$$

The real or imaginary areal specific areal capacitance (C', C'') are calculated by:

$$C' = -\frac{Z''}{2\pi f |Z|^2 A}$$
$$C'' = -\frac{Z'}{2\pi f |Z|^2 A}$$

τ_0 is derived from the frequency at maximum C''

$$\tau_0 = \frac{1}{f_0}$$

where f is the frequency, A is the electrode area, Z' and Z'' are the real and imaginary impedance, f_0 is the frequency at maximum C''.
